# Nigeria mental health law: Challenges and implications for mental health services

**DOI:** 10.4102/sajpsychiatry.v30i0.2134

**Published:** 2024-04-19

**Authors:** Gerald O. Ozota, Ruth N. Sabastine, Franklin C. Uduji, Vanessa C. Okonkwo

**Affiliations:** 1 Department of Pharmacy, Federal Neuropsychiatric Hospital, Yaba, Lagos, Nigeria; 2 Department of Pharmacy, Faculty of Pharmaceutical Sciences, University of Nigeria Nsukka, Nsukka, Nigeria; 3 Pharmacy Council of Nigeria, Abuja, Nigeria

**Keywords:** Nigeria, mental health law, mental health services, health policy, mental health advocacy

## Abstract

**Background:**

The Nigerian mental health law titled the *Lunacy Act* of 1958 has been under scrutiny for violating the human rights of people with mental illness. The call to reform the obsolete *Lunacy Act* has garnered attention from the government, as the law has been unamended for over 60 years.

**Aim:**

This study presents the challenges and implications of the new mental health law to the mental health services of Nigeria.

**Methods:**

ScienceDirect, PubMed, and Google Scholar were used to find pertinent material. The implications and difficulties facing the new mental health law examined from the literature were discussed. Recommendations were made following an exploratory search for literature on mental health legislation in Nigeria.

**Results:**

The new Law in Section 5(6) saw the introduction of mental health services in primary and secondary healthcare. It also addresses critical issues such as non-discrimination, fundamental human rights, standards of treatment, access to information, confidentiality and autonomy, and the employment rights of persons with mental health and substance abuse-related disorders. The Law failed to include mental health services in the country’s health insurance system.

**Conclusion:**

There is a need for legislation to meet people’s mental health needs and encourage them to seek treatments, such as regulations that protect against discrimination and harsh treatment of people with mental illness.

**Contribution:**

Nigerian mental health services would benefit from the new mental health law if the key issues raised in this review are addressed.

## Introduction

Nigeria, with a population of approximately 180 million, confronts a notable predicament concerning mental health, with an estimated 20% of its populace experiencing various forms of mental health issues.^1^ This substantial proportion translates to a considerable number of several million individuals grappling with mental health challenges within the country. Despite Nigeria’s categorisation as a low- and middle-income country (LMIC), aligning with the status of more than 80% of the global population, a mere fraction of less than 10%, of those affected can access appropriate mental healthcare and treatment.^2^ Tragically, a significant number of these individuals encounter notable obstacles in accessing requisite treatments, safeguarding their rights, securing rehabilitation services, or obtaining other essential support.^1^ This predicament underscores the persistent neglect of mental health as a crucial facet of healthcare in the Nigerian context. In Nigeria, the observable gap between the provision of healthcare services and the demand for mental health services is clearly evident. Despite notable strides in addressing pervasive public health concerns and striving towards an all-encompassing health policy, the country has encountered several obstacles in the area of mental health services. These challenges pertain to policy formation and enactment, financial allocation, research endeavours, educational initiatives, and the incorporation of mental healthcare within primary healthcare systems.^3^

In recent times, the recognition of mental health as a critical concern within the public health and developmental spheres has become apparent to the Nigerian government. This realisation has spurred the government to revise the mental health law that was initially introduced in 1916, subsequently bolstered by the introduction of the Mental Health Policy in 1991. Nigeria’s new mental health Act, ‘*National Mental Health Act*, 2021’, was signed into law by the president in January 2023.^4^ The Lunacy Ordinance, implemented in 1916, was Nigeria’s first mental health law. In 1958, the revision of the *Lunacy Ordinance to the Lunacy Act* allowed medical practitioners and judges to detain individuals with mental illnesses.^5^ After being renamed the *Lunacy Act of 1958*, this Act has since not been amended. A little over 60 years after passing the *Lunacy Act of 1958*, there has been a call to repeal the obsolete Act because of certain deficiencies such as the utilisation of the term ‘lunatic’ and ‘insane’ as descriptors for individuals who are experiencing mental illness; also, the Act exhibits a deficiency in adequately delineating the conceptual boundaries of mental disease. Moreover, the absence of a comprehensive and well-defined conceptualisation of mental disease poses a significant challenge in identifying individuals eligible for admission to receive appropriate therapeutic interventions, hence creating certain human rights issues.^6^

There is a preponderance of adversity in Nigerian society, which regularly threatens Nigerians’ mental health and well-being. Such adverse conditions include, among others, a high poverty rate, a high unemployment rate, economic and religious instability, traumatic events, human rights violations, and a deeply established belief in supernatural illness.^7^ This new Act is a big step forward for mental healthcare in Nigeria because it aims to improve and protect the lives of people with mental illnesses while also fixing the deficiencies of the *Lunacy Act* as earlier mentioned. It also shows how serious the government is about closing Nigeria’s substantial mental healthcare gap and, eventually, getting everyone covered by health insurance. This Act is timely for Nigerian mental health law and policy to safeguard persons with mental illness from egregious human rights abuses, such as humiliating treatment and abject poverty.

This article therefore aims to provide a critical assessment of the practical difficulties and obstacles encountered in the implementation and execution of the mental health law, as well as to evaluate the impact of these challenges on the overall provision of mental health services in Nigeria. By shedding light on the intricacies of Nigerian mental health law and its effects on mental health services, this review endeavours to offer insights that can inform policy recommendations and interventions aimed at enhancing the effectiveness and inclusivity of mental healthcare in the country.

## Methods

Google Scholar, PubMed, and ScienceDirect were used to retrieve research articles. These databases were searched using terms such as ‘mental health law and (or) policies and (or) Nigerian mental health legislation’. With this search strategy, pertinent literature was chosen. The authors evaluated the quality of the article concerning the subject of our review based on the articles’ contents. Using the selected articles, previous laws and their flaws were examined. The implications and challenges the new mental health law posed were examined in the literature, and recommendations were given.

### Ethical considerations

The study protocol was granted ethical clearance waiver NHREC/05/05/2022B-FWA00002458-IRB00002323 by the Health Research and Ethics Committee (HREC) of the University of Nigeria Teaching Hospital (UNTH), Ituku-Ozalla, Enugu State as the study required no human or clinical sample and since no human participant were required for the study.

## Discussion

### Mental health legislation in Africa

Owing to limited mental health resources and the growing emphasis on institutional support for citizens’ well-being in LMICs, the prevalence of mental disorders in LMICs in Africa is of particular concern.^8^ Mental, neurological, and substance use (MNS) diseases are a significant problem in Africa, affecting both children and adults alike.^9^ With the inclusion of mental health into the Sustainable Development Goals (SDGs) for 2015, a worldwide commitment was made to include mental health among the highest investment priorities as a health, humanitarian, and socioeconomic focus.^10^ Misconceptions about mental illness often foster stigma and poor attitudes towards individuals with such diseases. Such beliefs are widespread in sub-Saharan African societies, leading to the persecution and even mutilation of those with mental health conditions.^2^ Curiously, persons with professional health education backgrounds, such as nurses and physicians, often hold beliefs attributing the causes of certain mental illnesses to witchcraft.^11^

In Africa, 64% of nations lack mental health laws or have outdated legislation that does not effectively protect the rights of individuals with mental disorders.^4^ According to the World Health Organization (WHO) Mental Atlas (2022):

A mental health policy is an official statement by a government that defines a vision with a set of values, principles, and objectives and an overall plan of action to achieve that vision and improve the mental health of a population.^12^ (p. 26)

In Africa, laws are shaped by distinctive historical, sociopolitical, and contextual considerations. Therefore, mental health law in Africa does not fully reflect either logical philosophical arguments or the numerous Resolutions and Declarations by international organisations.^13^ Mental health laws in countries such as Uganda, South Africa, Sierra Leone, and Gambia still allow for the involuntary admission of people with mental health illnesses^14,15,16^ while Ghana makes certain reservations as only specialised facilities are empowered to admit patients against their will.^14^ Although the existing *Mental Health Care Act* 17 of 2002 in South Africa has significantly enhanced the treatment process by enabling involuntary treatment with more consideration for patients’ civil liberties, it remains in conflict with the United Nations 2006 Convention on the Rights of Persons with Disabilities and its subsequent interpretations.^14^ Generally, mental health legislations in Africa still accord inadequate emphasis on the human rights of those with mental illness. According to the Mental Health Atlas 2020, only 49% of African Member States have updated mental health laws.^12^ As of 2020, out of the 71% of the nations in the African Region that have mental health legislation or a framework, only 14% have fully implemented it.^17^ Nevertheless, there are still calls to make these laws encompassing as only 29% of these nations have child and adolescent mental health (CAMH) policies and strategic plans.^9^

A policy brief entitled ‘Developing Effective Mental Health Laws in Africa’ issued in 2009, elucidates pertinent concerns pertaining to the mental health legislation in four African nations.^18^ The primary emphasis lies on the issue of incapacity and forced treatment, while there is limited advocacy for voluntary treatment predicated on the principles of autonomy and informed consent. The laws frequently fail to adequately acknowledge the significance of community-based care and lack provisions for the prevention of involuntary hospitalisation or promotion of principles of respect, dignity, autonomy, and non-discrimination for individuals with mental illnesses. The policy brief strictly recommended a revisit and modification of the various mental health laws to adhere to international standards.

### The Nigerian National Mental Health Act

There have been several efforts to formulate a new mental health Act in Nigeria that would reflect the global standard of management of mental illness. The first notable effort made to amend the lunacy law under democratic rule was in 2003. These efforts stem from the recommendation of Nigerian National Mental Health Policy of 1991. Within this period, the Nigerian Senate introduced the Mental Health Bill, propelled by two incumbent senators, both esteemed medical practitioners, with one serving as a psychiatrist. Encouragingly, the bill successfully passed its initial reading in the Senate. In adherence to the legislative process in Nigeria, bills necessitate three readings and presidential endorsement to be enacted. Regrettably, because of the expiration of the Senate’s term in 2009 and the primary sponsor’s death, the bill encountered delays between the first and second readings.

According to the Nigerian National Mental Health Policy of 1991, mental health should be integrated with all levels of general medical services.^17^ The 2013 Policy reaffirms this dedication to providing mental health services in primary care through the ‘Mental Health Services Delivery and the National Mental, Neurological, and Substance Use Programme and Action Plan for Nigeria’.^19^ Primary care and municipal authorities were assigned a significant part of the responsibility for ensuring adequate mental healthcare delivery under the programme.^20^ Despite all the effort placed into these policies, they have not yet been fully implemented. Initiatives for national mental health reform in 2003 and 2013 were unsuccessful.^21^

Within the framework of the National Policy for Mental Health Services Delivery, an additional bill emerged in 2013, and faced abandonment because of insufficient backing. Subsequently, in 2019, a proposed legislation, titled ‘A Bill for an Act to provide for the establishment and regulation of mental health and substance abuse services’, aimed at safeguarding individuals with mental health needs and instituting the National Commission for Mental and Substance Abuse Services for efficient mental health management in Nigeria. This bill underwent successful passage during its second reading and underwent a public hearing in 2020. Ultimately, the Mental Health Bill 2021 gained approval from the National Assembly on 28 November 2022, subsequently advancing to the president for endorsement. On 05 January 2023, President Muhammadu Buhari ratified the bill into law.

The National Mental Health Act No. 46 of 2021 is:

[*A*]n act to provide for the enhancement and regulation of mental health and substance abuse services; to protect persons with mental health needs, and to establish a national commission for mental and substance abuse services for the effective management of mental health in Nigeria and other related matters. (A. 1490)

It is divided into five parts and addresses the rights of people with mental illnesses, particularly during their stay and therapy in mental health facilities.

### Implications and challenges of the new mental health Act

#### Mental health integration into primary healthcare

Nigeria’s healthcare system has made significant strides in recent years, with a three-tiered system comprising primary healthcare available mainly in rural areas, secondary healthcare provided by state governments, and tertiary healthcare overseen by the federal government. Mental health services have traditionally been restricted to tertiary institutions, with less than 300 psychiatrists catering to Nigeria’s vast population, resulting in limited accessibility to mental health services. The Nigerian National Mental Health Policy of 1991 stated that mental health should be integrated into all levels of general health services.^22^ But this has been invisible for several decades up to the present time. In Section 24(5) of the new Act, the introduction of mental health services in both primary and secondary healthcare is now legalised. It states:

Notwithstanding subsection (1), the Minister shall ensure that the existing facilities at the primary, secondary, and tertiary levels of healthcare are effectively utilized for the purpose of the implementation of this Act. (A. 1417)

This was a follow-up to Section 24 (1) that authorises the utilisation of existing public health facilities:

Every public healthcare facility shall make provision for integrated mental health treatment at all levels in line with the guidelines established by the Department for the purposes of effective implementation of the provisions of this Act. (A.1416)

This is a right step in the right direction as many studies have assessed the effectiveness of primary care integration of mental health in LMICs.^17^ In their 2001 report on world health, the WHO recommended that mental health services be administered through the primary care system to increase social acceptance, broaden availability, and cut down on other expenses such as those associated with travel.^23^

#### Human rights of individuals with mental health and substance addiction disorders

The *Lunacy Act* gave magistrates and medical practitioners the power to detain someone who has a mental illness. The Act came under significant criticism as Nigeria was a pact to numerous international treaties relating to human rights. The new mental health law addresses critical issues such as non-discrimination, fundamental human rights, standards of treatment, access to information, confidentiality, and autonomy, and the employment rights of persons with mental health and substance abuse-related disorders. Section 12(1) of this law states:

Without prejudice to the provisions of this Act, persons in need of mental and substance abuse services, irrespective of the cause, nature or degree of past or present mental health conditions, shall – (1) have the same fundamental rights as a fellow citizen; and (2) not be subjected to any form of discrimination. (A. 1413)

The law in this section also ensures non-discrimination of people based on physical disability, age, gender, race, language, religion, ethnicity, or nationality. Also, it may include the right to education, vocational training, leisure, recreational activities, full employment, and participation in civil, economic, social, cultural, and political activities. This section also confers the entitlement to a legal practitioner on a patient. When a patient cannot afford such, the ‘Legal Aid Council of Nigeria or the National Human Rights Commission’ is mandated to provide legal assistance. The legislation guarantees a person with a mental health condition the right to freely and voluntarily consent to treatment or care, and this should be recorded in the patient’s clinical file. Section 4 of the law states that ‘A person may make an application to a court for the involuntary admission and treatment of a person believed to be suffering from severe mental disorder’. Aluu and Caldas-de-Almeida criticise Section 30(4) of the law, pointing out that one of the prerequisites for a medical suggestion to a court for temporary involuntary treatment is a lack of competence to give informed permission, and it can lead to misinterpretation. However, they agreed that despite the loopholes in this law, it has a positive prospect of changing mental health services in the country as it has seen an improvement from the previous legislation.^24^

#### Subsidy of mental health services

According to the WHO, government spending on mental health in Nigeria accounts for barely 4% of all health spending. Psychosis, bipolar illness, and depression treatment are not covered by national health insurance or reimbursement programmes.^21^ The law failed to include mental health services in the country’s insurance system. The National Health Insurance Scheme’s (NHIS) goal is to lower out-of-pocket expenses in all forms because doing so increases access to healthcare for all people, especially people with low incomes. Since its tripartite public–private operation, which consisted of the NHIS, health maintenance organisations (HMOs), and healthcare providers, began in 2005,^25^ less has been seen about the inclusion of mental health services as it pertains to other health services. In order to deliver high-quality and inexpensive healthcare, many responsible governments assimilate social health insurance as a governance obligation. In addition to being high-quality and affordable, healthcare must have universal coverage to increase accessibility. Financial constraints have often limited people’s access to mental health services. Because of its poor budgetary allocation, the government has failed to provide subsidised healthcare for all, especially regarding mental health. Given the strong ties between poverty and mental illness, it is concerning that reliance on out-of-pocket disbursements discourages the utilisation of mental health treatments by individuals with limited earnings. A study by Ajefu et al. demonstrated that financial inclusion improves mental health and reduces the probability of presenting symptoms of depression.^26^

#### Interprofessional rivalry

The law did not consider interprofessional rivalry, which can arise because of allocating roles. Healthcare providers have a significant responsibility for their patients’ health and safety. However, competition among these service providers has grown because of factors such as organisational structure, specialisation, and the diversity of skill sets. While rivalry can result in health competition, it can sometimes lead to tension. Studies indicate that conflict is substantially more prevalent in the healthcare industry because of frequent and varied interactions among healthcare workers.^27^ Doctors, pharmacists, nurses, medical laboratory scientists, and other healthcare workers with various areas of specialisation must collaborate to provide healthcare services. However, conflict is unavoidable whenever these healthcare professionals collaborate as a team, and it can negatively impact patient care, job happiness, personal wellness, and professional productivity. This conflict stems from the fact that some professions seem alienated from managerial roles or are exempted from some benefit, which their fellow workers enjoy. For example, in Nigeria, only a consultant physician can head a hospital as the medical director or the chief medical director. This has come under criticism as other healthcare providers see this as discrimination. Nwobodo et al. noticed that this rivalry is mainly based on management and administrative roles.^28^ In Section 6(2) the new law enacted the creation of a governing board of the commission, which consists of the chief executive and/or executive secretary, who must be a consultant psychiatrist, and presidents of the associations of psychiatrists, psychiatric nurses, clinical psychologists, medical social workers, and occupational therapists. The exemption of other healthcare practitioners who actively take part in the care of the patients with mental illness, such as radiographers, pharmacists, and medical laboratory scientists, can create a feeling of neglect. The outcome of this can affect patient care and the knowledge these healthcare workers can contribute to the board is not utilised. In the appointment of the members of the mental health tribunal in Section 12(2), healthcare workers such as radiographers, pharmacists, and medical laboratory scientists were also exempted. It further reinforces the relevance of, and the necessity for, legislative measures that encourage harmonious relationships among healthcare personnel. If this goal is met, the Nigerian healthcare industry will be brought up to international standards faster and healthcare workers in Nigeria will be more dedicated to their jobs.

### Recommendation

Nigeria’s 2021 Mental Health Act represents a significant improvement from the outdated Lunacy Act.^29^ It provides a comprehensive framework for providing mental health services and protecting the rights of people with mental illness. Nonetheless, despite these developments, a few key areas still need enhancement. To begin, there is a need for increased funding in the mental health sector to address the current inadequacies in service delivery, facilities, and research. The government should increase budgetary allocations to the mental health sector, seek support from donor agencies, and partner with the private sector to invest in mental health services. In addition, all Nigerians, regardless of their financial situation, should have access to and be able to purchase mental healthcare by including mental healthcare in the NHIS. This would lessen the financial strain on patients and their families while ensuring that individuals who require mental health services can access them. Finally, there needs to be no competition among mental health professionals. Better care for people with mental health and substance misuse issues can be promoted by ensuring that all relevant healthcare professions, pharmacists, medical laboratory scientists, radiographers, and others are represented on the commission’s governing board. The inclusion of diverse representation within the commission guarantees that a wide range of expertise is taken into account, resulting in the development of comprehensive and efficient solutions that address the complex and multifaceted aspects of mental health and drug use problems.

## Conclusion

Governments worldwide and the Nigerian government in particular, are being put to the test by the staggering toll that mental illness is taking on populations. There is a great need to put in place laws that will cater to the mental health needs of people and also make it attractive for people to seek these services, such as laws that will prevent discrimination and inhumane treatment of people with mental illness. The new mental health law holds an excellent prospect for Nigerian mental health services as it addresses critical issues that revolve around human rights, involuntary treatment, and detention of people with mental health issues. While waiting for its full implementation, there is a call to give a cursory look at the inclusion of mental health services in the country’s health and also to make an inclusion of other health workers in the administrative roles prescribed in the law to prevent interprofessional rivalry.
